# Effect of Acetaminophen Ingestion on Thermoregulation of Normothermic, Non-febrile Humans

**DOI:** 10.3389/fphar.2016.00054

**Published:** 2016-03-14

**Authors:** Josh Foster, Alexis Mauger, Katie Thomasson, Stephanie White, Lee Taylor

**Affiliations:** ^1^Applied Sport and Exercise Physiology Research Group, Institute of Sport and Physical Activity Research, Department of Sport Science and Physical Activity, University of BedfordshireBedfordshire, UK; ^2^Endurance Research Group, School of Sport and Exercise Sciences, University of KentChatham, UK; ^3^ASPETAR, Qatar Orthopaedic and Sports Medicine Hospital, Athlete Health and Performance Research CentreDoha, Qatar

**Keywords:** acetaminophen, paracetamol, tylenol, thermoregulation, temperature, therapeutic hypothermia

## Abstract

In non-febrile mouse models, high dose acetaminophen administration causes profound hypothermia. However, this potentially hazardous side-effect has not been confirmed in non-febrile humans. Thus, we sought to ascertain whether an acute therapeutic dose (20 mg⋅kg lean body mass) of acetaminophen would reduce non-febrile human core temperature in a sub-neutral environment. Ten apparently healthy (normal core temperature, no musculoskeletal injury, no evidence of acute illness) Caucasian males participated in a preliminary study (Study 1) to determine plasma acetaminophen concentration following oral ingestion of 20 mg⋅kg lean body mass acetaminophen. Plasma samples (every 20 min up to 2-hours post ingestion) were analyzed via enzyme linked immunosorbent assay. Thirteen (eight recruited from Study 1) apparently healthy Caucasian males participated in Study 2, and were passively exposed to 20°C, 40% r.h. for 120 min on two occasions in a randomized, repeated measures, crossover design. In a double blind manner, participants ingested acetaminophen (20 mg⋅kg lean body mass) or a placebo (dextrose) immediately prior to entering the environmental chamber. Rectal temperature, skin temperature, heart rate, and thermal sensation were monitored continuously and recorded every 10 min. In Study 1, the peak concentration of acetaminophen (14 ± 4 μg/ml) in plasma arose between 80 and 100 min following oral ingestion. In Study 2, acetaminophen ingestion reduced the core temperature of all participants, whereas there was no significant change in core temperature over time in the placebo trial. Mean core temperature was significantly lower in the acetaminophen trial compared with that of a placebo (*p* < 0.05). The peak reduction in core temperature in the acetaminophen trial was reached at 120 min in six of the thirteen participants, and ranged from 0.1 to 0.39°C (average peak reduction from baseline = 0.19 ± 0.09°C). There was no significant difference in skin temperature, heart rate, or thermal sensation between the acetaminophen and placebo trials (*p* > 0.05). The results indicate oral acetaminophen reduces core temperature of humans exposed to an environment beneath the thermal neutral zone. These results suggest that acetaminophen may inhibit the thermogenic mechanisms required to regulate core temperature during exposure to sub-neutral environments.

## Introduction

Acetaminophen (APAP) is a widely used analgesic antipyretic drug branded as Tylenol^®^ in North America and Paracetamol in Europe. It is available over-the-counter in various single-entity formulations and is commonly prescribed in combination with various opioids to manage moderate pain. Despite its well-established validity as an analgesic and antipyretic via inhibition of cyclooxygenase (COX; Aronoff et al., 2006; Li et al., 2008), a growing body of evidence also demonstrates a third, hypothermic action of APAP, namely core temperature (T_C_) reduction in the absence of fever. This side effect may be beneficial for inducing therapeutic hypothermia, but may also increase rates of accidental hypothermia (via exacerbated T_C_ reductions in environments beneath thermal neutrality; Foster et al., 2015). 4°C reductions in T_C_ have been shown within rodents following intravenous APAP administration (Li et al., 2008; Ayoub et al., 2011; Gentry et al., 2015). However, such a hypothermic T_C_ response following oral ingestion of APAP has not been confirmed in non-febrile humans, within a controlled laboratory environment.

Confirming if APAP exhibits a hypothermic action in humans is pertinent. Firstly, due to the inverse relationship between T_C_ and neurological outcome after stroke or traumatic brain injury (Reith et al., 1996), APAP could be used as a cheap, safe, and easily administered pharmacological method to reduce T_C_ when more invasive and logistically challenging methods are not available or appropriate (den Hertog et al., 2009). Indeed, APAP has been shown to reduce mean T_C_ in non-febrile stroke patients (Kasner et al., 2002), however, the APAP dose used was small (650 mg), T_C_ was only averaged across a 24 h period, and there were no obvious environmental or nutritional controls in place. Secondly, if APAP reduces the resting T_C_ of healthy humans, it may be involved in the pathology of accidental hypothermia. For example, if APAP reduces T_C_ via increasing heat loss or decreasing heat production, this places individuals who consume APAP at greater risk of developing hypothermia, especially in winter months. This is of particular concern to thermoregulatory vulnerable individuals, i.e., the very young (aged 0–4 years) or the elderly (age ≥ 65 years), who together contributed ≥85% of UK hospital admissions in 2014 where hypothermia was the primary or secondary diagnosis (HSCIC, 2015). As APAP is the most frequently administered over-the-counter medication worldwide (Blieden et al., 2014), any hypothermic action could have deleterious implications for a significant number of people, as outlined above.

The aim of the present study was to determine whether acute, oral APAP ingestion alters thermoregulatory control during passive exposure to a sub neutral environment of 20°C. It was hypothesized that APAP would decrease T_C_ without any change in skin temperature (T_SK_), thermal sensation or heart rate.

## Materials and Methods

### Ethical Approval

All experimental procedures were approved by the University of Bedfordshire’s Institute for Sport and Physical Activity Research Ethics committee (approval code 2012ASEP021), and they conformed to the standards set by the World Association Declaration of Helsinki ‘Ethical Principles for Research Involving Human Subjects’.

### Participants

**FIGURE 1:**
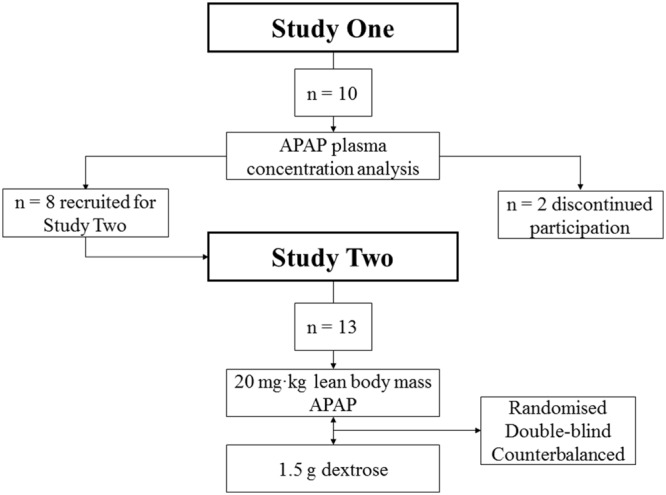
**Trial profile.** APAP, acetaminophen.

Ten Caucasian males [Age (23 ± 2 years), Height (181 ± 8 cm), Mass (80 ± 8 kg), body fat (16.1 ± 4)] participated in Study 1. Thirteen Caucasian males [Age (23 ± 1 years), Height (174 ± 3 cm), Mass (73.6 ± 8 kg), body fat (15.5 ± 4.8%)] participated in Study 2. Eight participants from Study 1 went on to participate in Study 2, with two discontinuing their participation for Study 2 (see **Figure 1** for trial profile). Participants were provided with written information regarding all experimental procedures, with supporting oral explanations from the principal investigator. Participants then subsequently provided written informed consent. The participants were non-smokers, non-febrile (resting T_C_ < 38°C), and were free from musculoskeletal injury.

### Inclusion/Exclusion Criteria

Prior to each laboratory visit, participants completed an informed consent sheet, alcohol use disorder identification test (AUDIT; Saunders et al., 1993) a breathalyzer test (AlcoSense, One, Berkshire, UK), and an APAP risk assessment questionnaire. To avoid the risk of liver damage inflicted by APAP, participants were not able to participate in the research if they scored above ten on the AUDIT questionnaire or alcohol was present in their bloodstream. No participants presented with any pre-existing medical conditions that may have put them at an increased risk of APAP toxicity. Due to potential thermoregulatory adaptions (Gibson et al., 2015), individuals were not permitted to take part in any experimental procedures if they were heat acclimated or acclimatized. Thus, those who had traveled to a hot climate or participated in a laboratory based heat acclimation protocol less than three weeks prior to the experiment were not permitted to take part (Garrett et al., 2011). All participants presented with a stable core temperature (36.5–37.5°C) and were thus considered non-febrile.

## Study 1

### Study 1 Design

To identify when the peak plasma concentration of ACT arises after oral ingestion (20 mg⋅kg LBM), eight Caucasian males visited the laboratory on one occasion. Participants adhered to all experimental controls listed in the “general experimental controls” section.

### Study 1 Controls

On the trial day, APAP (Paracetamol, Aspar Pharmaceuticals, London, UK) was administered at a dose equal to 20 mg⋅kg of lean body mass. Each capsule contained a maximum of 500 mg of APAP. The dosing range of APAP in the present work was 1019–1420 mg (mean = 1293 ± 163 mg). This dosing was advised by a leading clinician and consultant anesthetist, and has been used previously (Mauger et al., 2014; Coombs et al., 2015). All participants arrived at the laboratory having fasted overnight (from 0000 to arrival). To control the gastric emptying rate of APAP, participants ingested a standardized meal [cornflakes (50 g), milk (250 ml) and 1 l of water] 1 h prior to APAP or placebo ingestion. Experimental trials took place within a custom built environmental chamber (Custom build, T.I.S.S, Hampshire, UK) which simulated the desired environmental condition of 20°C and 40% relative humidity. The participants’ clothing was standardized across all trials, in which they were barefoot, topless, and wore knee length shorts.

### Study 1 Protocol

Participants arrived at the laboratory at 0830. Upon arrival, participant’s lean body mass was calculated via air displacement plethysmography (Bod Pod, 2000A, Birmingham, UK). At 0845 participants consumed a standardized meal (see “Study 1 Controls”). At 0920, participants remained seated and a 20G cannula (Introcan^®^ Safety Winged, B Braun Medical, Sheffield, UK) was placed in a prominent vein within the antecubital fossa. At 0930, participants entered the environmental chamber (20°C, 40% r.h.). At 0945, participants orally ingested APAP (Paracetamol, Aspar Pharmaceuticals, London, UK). Blood samples were drawn into a heparin coated EDTA tube (Vacuette^®^, Greiner Bio-One, Stroudwater, UK) immediately prior to APAP ingestion, and subsequently at 20, 40, 60, 80, 100, and 120 min post ingestion. Peak plasma levels of APAP were quantified using a commercially available ELISA kit (Microplate EIA kit, Alere toxicology, Abingdon, UK) read in duplicate at a dual wavelength of 450 and 650 nm (Sunrise, Tecan, Seestrasse, Männedorf). The average inter-plate CV was 5% (intra-plate CV not applicable due to assay layout). Venous hemoglobin (Hb 201+, Hemocue, Staines, UK) and haematocrit (Haematospin 1300, Hawksley, Sussex, UK) concentrations were taken in duplicate and averaged at each point of blood sampling. This data was used to calculate changes in plasma volume according to the Dill and Costill method (Dill and Costill, 1974), and APAP concentrations were subsequently adjusted in accordance with any changes in plasma volume.

## Study 2

### Study 2 Design

To determine the hypothermic effect of APAP, 13 participants visited the laboratory at the same time of day on two occasions, each separated by at least 7 day. Both visits were randomized and double blinded. The trials took place within an environmental chamber (20°C, 40% r.h.) set beneath the thermoneutral zone (i.e., subneutral; Kingma et al., 2014; Schlader, 2015) for 120 min. A sub-neutral environment was chosen for two reasons, (i) to ensure that heat loss mechanisms would not become activated during the protocol and (ii) to help replicate the sub-neutral conditions in previous animal work concerning APAP induced hypothermia (Botting and Ayoub, 2005).

### Study 2 Controls

On each trial day, APAP (Paracetamol, Aspar Pharmaceuticals, London, UK) or a placebo (dextrose, MYPROTEIN, Cheshire, UK) was administered at a dose equal to 20 mg⋅kg of lean body mass. Each capsule contained a maximum of 500 mg of APAP. The dosing range of APAP in the present work was 1019–1420 mg (mean = 1226 ± 135 mg). Controls for gastric content, nutritional intake, environmental conditions, and clothing were identical to that of Study 1 (see “Study 1 Controls”).

### Study 2 Protocol

Participants arrived at the laboratory at 7000 or 1000, where each participant’s time of arrival was consistent through all experimental trials to account for any circadian rhythm or diurnal variations in T_C_ (Waterhouse et al., 2005). Upon arrival (0700 or 1000), participants were instrumented for the measurement of T_C_, T_SK_, and heart rate (see “Instrumentation and Equations” for details). Thirty min after arrival (0730 or 1030), participants consumed the standardized breakfast. Sixty min after the meal was consumed (0830 or 1130), participants ingested APAP or placebo. Participants remained rested in an upright-seated position between meal consumption and APAP or placebo ingestion, and for the duration of data collection to ensure resting physiological status was attained and maintained throughout the data collection. Data collection began immediately after ingestion of APAP or placebo, i.e., 60 min after meal consumption. Resting measurements of T_C_, T_SK_, heart rate, and thermal sensation were taken 5 min prior to APAP or placebo ingestion (0825 or 1125), and subsequently every 10 min for 120 min post ingestion.

### Study 2 Instrumentation and Equations

Copper based thermocouples (Grant, EUS-U-VS5-0, Dorset, UK) connected to a wireless data logger (Grant, Squirrel Series, Dorset, UK) recorded *T*_SK_ at four sites: calf, thigh, chest, and triceps (Bruning et al., 2013). Thermocouples were securely attached to the belly of each muscle by hypafix surgical adhesive tape (BSN medical, D-22771, Hamburg, Germany). The weighted T_SK_ of four sites was subsequently calculated using the equation (Ramanathan, 1964) below:

$$T_{\mathrm{SK}}=0.3*(T_{\mathrm{arm}}+T_{\mathrm{chest}})+0.2*(T_{\mathrm{calf}}+T_{\mathrm{thigh}})$$

T_C_ was measured via insertion of a rectal thermistor (Henleys, 400H/4491H, Hertfordshire, UK) 10 cm beyond the anal sphincter. The thermistor was connected via cable to a portable data logger (Libra Medical, ET402, Birmingham, UK), in which T_C_ was continuously displayed throughout each experimental protocol.

Thermal sensation (Young et al., 1987) was obtained using a 0–8 scale ranging from unbearably cold (0) to unbearably hot (8). Heart rate was measured during all tests using short-range telemetry. A Polar heart rate transmitter belt (Polar, FS1, Birmingham, UK), coated with conductive gel to enhance signal detection, was strapped to the participant’s chest. Heart rate was displayed on a corresponding Polar watch (Polar, FS1, Birmingham, UK).

### Statistical Analysis

Statistical analyses were performed using IBM SPSS statistics version 21 (SPSS Inc., Chicago, IL, USA). Power analyses were conducted with GPower software version 3.1 (Heinrich University, Düsseldorf, Germany). Utilizing T_C_ data from a previous experiment where APAP was tested as a hypothermic agent (Dippel et al., 2003a), it was determined that a total of 13 participants were required to achieve a statistical power of 90%. Statistical assumptions were checked using conventional graphical methods (quantile–quantile plots, histograms; Grafen and Hails, 2002) and were deemed plausible. Central tendency and dispersion are reported as means ± SD. Mean differences in T_C_, T_SK_, heart rate, and thermal sensation between APAP and placebo were measured using a two-way analysis of variance (ANOVA). In the event of a significant *F* statistic for the main effects or interaction effects, a Sidak *post hoc* adjustment was performed, and 95% confidence intervals (CI) are presented where appropriate. The two-tailed alpha level of significance testing was set as *p* < 0.05.

## Results

### Study 1

**FIGURE 2:**
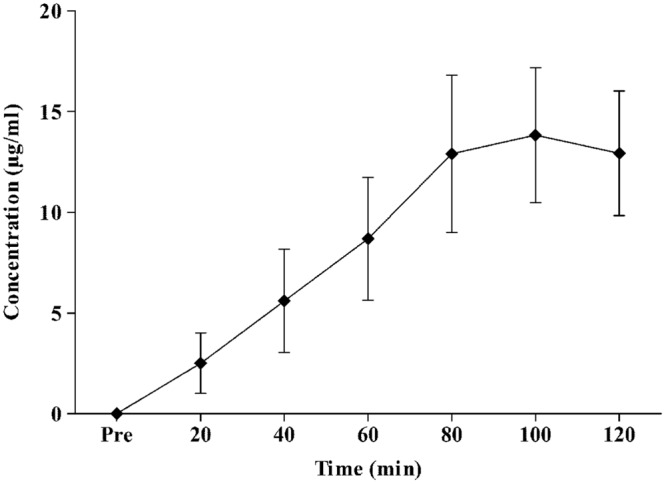
**Mean ± standard deviation values for plasma APAP concentration response to oral intake of 20 mg⋅kg lean body mass-1 APAP during a resting two-hour period**.

The peak plasma concentration of APAP during the 120 min experiment was 14 ± 4 μg/ml (range, 8–19 μg/ml). The time for APAP to reach maximal concentrations was 96 ± 13 min (range, 80–100 min). **Figure 2** displays the plasma concentration response of APAP, every 20 min for 2 h.

### Study 2

#### T_C_

**FIGURE 3:**
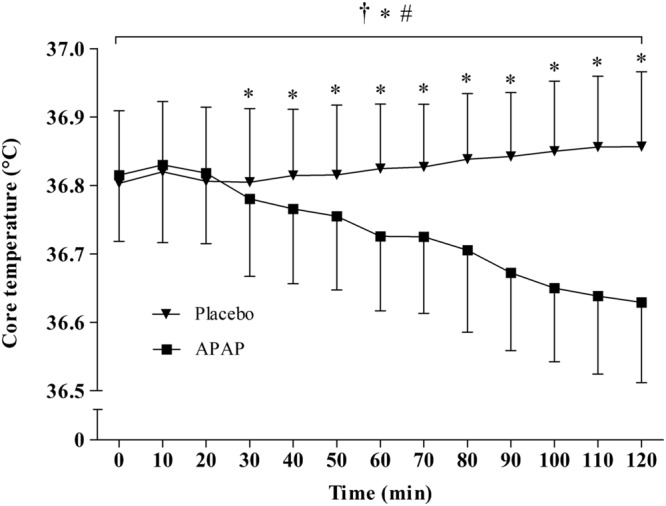
**Mean ± standard deviation values for core temperature (T_C_) in both the APAP and placebo conditions.** ^∗^Significant main effect for condition. ^#^Significant main effect for time. ^†^Significant interaction effect.

**FIGURE 4:**
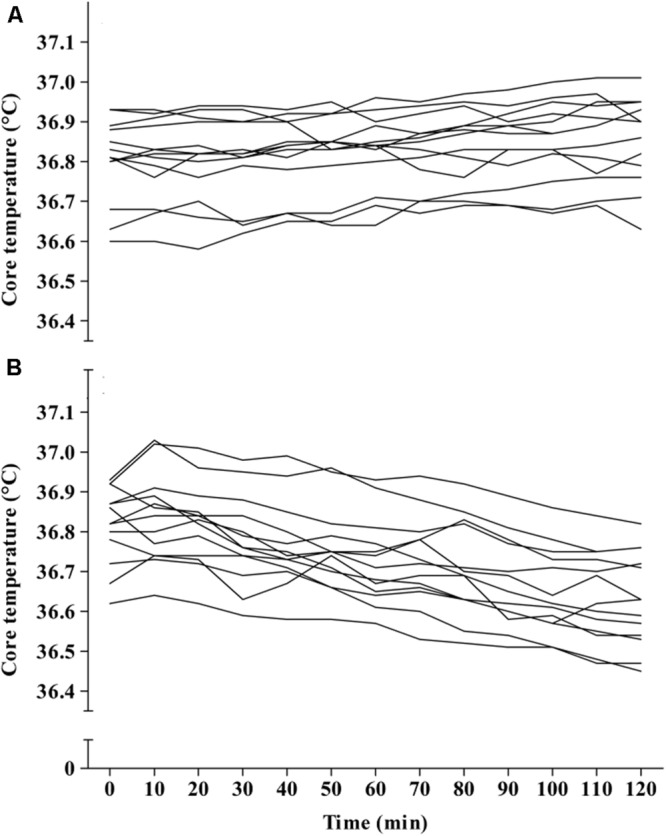
**Individual core temperature (T_C_) responses in the placebo **(A)** and APAP condition (B)**.

Main effects were found for condition (*F*_1,1_ = 28.68, *p* < 0.001), and time (*F*_1,12_ = 17.23, *p* < 0.001) between APAP (36.73 ± 0.1°C; 95% CI = 36.67–36.8°C) and placebo (36.83 ± 0.1°C; 95% CI = 36.77–36.89°C). A significant interaction effect was also found (*F*_1,12_ = 51.68, *p* < 0.001), revealing that mean T_C_ was significantly lower in the APAP group from 30 min to the cessation of the trial. The peak T_C_ reduction from baseline arose 110 min (six subjects) or 120 min (seven subjects) after ingestion, and ranged from 0.1–0.39°C (mean = 0.19 ± 0.09°C). The T_C_ response to APAP ingestion is displayed in **Figure 3**. Individual responses to APAP are also shown in **Figure 4**.

#### T_SK_

There were no significant main effects for condition (*F*_1,1_ = 0.01, *p* > 0.05) between APAP (26.8 ± 1.3°C; 95% CI = 26–27.6°C) and placebo (26.9 ± 0.8°C; 95% CI = 26.41–27.3°C), but there was a main effect for time (*F*_1,12_ = 30.46, *p* < 0.001). No interaction effects were found (*F*_1,12_ = 0.39, *p* > 0.05).

#### Thermal Sensation

There were no significant main effects for condition (*F*_1,1_ = 0.37, *p* > 0.05) between APAP (3.4 ± 0.2; 95% CI = 3.4–3.5) and placebo (3.4 ± 0.2; 95% CI = 3.3–3.5), but there was a main effect for time (*F*_1,12_ = 17.76, *p* < 0.001). No interaction effects were found (*F*_1,12_ = 0.341, *p* > 0.05).

#### Heart Rate

There were no significant main effects for condition (*F*_1,1_ = 0.76, *p* > 0.05) between APAP (62 ± 8 b⋅min^-1^; 95% CI = 58–66 b⋅min^-1^) and placebo (63 ± 7 b⋅min^-1^; 95% CI = 60–66 b⋅min^-1^), but there was a main effect for time (*F*_1,12_ = 5.57, *p* < 0.001). No interaction effects were found (*F*_1,12_ = 0.24, *p* > 0.05).

## Discussion

Study 1 determined that APAP peak plasma concentration, following oral administration at 20 mg⋅kg of lean body mass, was 14 ± 4 μg/ml, with maximal concentrations reached 96 ± 13 min (range, 80–100 min; **Figure 2**) post ingestion. Study 2 demonstrated, in line with the stated hypothesis, that oral APAP ingestion, at the same dose as Study 1, elicited a notable hypothermic action in non-febrile humans. On average, T_C_ was 0.14°C lower during the 120 min exposure to 20°C in the APAP condition compared with a placebo. The maximum (peak) reduction in T_C_ in the APAP condition was 0.19 ± 0.09°C (range = 0.1–0.39°C). Having ingested APAP, all participants displayed a gradual decrease in T_C,_ and in seven participants, this did not plateau in the 120 min study period (**Figure 4**). However, due to the relatively short experiment time, the notion that T_C_ began to recover before the end of the 120 min period in the remaining six participants is speculative. To our knowledge, this is the first study that accurately demonstrates oral APAP ingestion to reduce non-febrile human T_C_ in sub neutral conditions (i.e., beneath thermal neutrality) within apparently healthy human participants (**Figure 3**).

The effect of APAP on non-febrile temperature regulation has been investigated previously in mice (Ayoub et al., 2004; Li et al., 2008; Ayoub et al., 2011; Gentry et al., 2015) and humans (Kasner et al., 2002; Dippel et al., 2003b; den Hertog et al., 2009). There are also reports of severe hypothermia (T_C_ = 28°C on hospital admission) following acute APAP overdose (Rollstin and Seifert, 2012). Prior to the present work, this potentially hazardous side-effect had not been confirmed in passive, nutritionally controlled participants or conducted in a temperature controlled environmental chamber. Despite a significant interaction effect (condition^∗^time) for T_C_, the 120 min trial period did not consistently allow for a plateau in T_C_ to be demonstrated, i.e., the maximum T_C_ reduction in seven of the thirteen participants was not seen prior to the final time point of 120 min (**Figure 4**). Although T_C_ seemed to plateau in six participants, a longer experimental duration is required to reliably determine when T_C_ will begin to recover to normal (i.e., pre APAP ingestion) values after administration of APAP. However, given that the cellular target of APAP for inducing hypothermia is not known in humans, it is difficult to predict if the peak reduction in T_C_ is line with the peak plasma or cerebral spinal fluid (CSF) concentrations.

Due to its worldwide use, the pharmacokinetics of APAP has been investigated extensively. However, it was important to determine the short-term concentration response in the present experiment (Study 1) as this has not been analyzed (i) following doses of 20 mg⋅kg of lean body mass and (ii) following the implemented nutritional controls. The data presented here is; however, in line with previous work. Early work using gas chromatography demonstrated that in adult humans (fasted and apparently healthy), oral doses of 1000 and 2000 mg APAP reach peak plasma concentrations in ∼60 and 120 min, respectively (Rawlins et al., 1977). An oral dose relative to lean body mass was used in the present experiment because the volume of distribution of hydrophilic drugs correlates more strongly with lean body mass compared with total body mass (Morgan and Bray, 1994). Consequently, the doses administered here ranged from 1019 to 1415 mg (mean = 1226 ± 135 mg). Thus, modeling previous pharmacokinetic data (Rawlins et al., 1977) and that from Study 1, it can be predicted that during Study 2, peak plasma concentrations were reached within the study period of 120 min. However, given that T_C_ did not consistently recover in the 120 min study period (**Figure 4**), this raises the notion that the peak reduction in T_C_ is more likely to be in line with peak CSF concentrations of APAP, which may take 4 hours to arise after an acute 1000 mg dose (Singla et al., 2012). Future work should elucidate when T_C_ begins to recover after acute APAP exposure, as this may have important implications if APAP is to be used as a hypothermic agent following brain injury (Saxena et al., 2015). It would also be beneficial to ascertain if the maximal T_C_ reductions are in line with peak CSF concentrations, as this would help identify if APAP induced hypothermia is mediated within the central nervous system.

In the UK, cold-related mortality presently accounts for at least one order of magnitude more deaths than heat-related mortality (around 61 and 3 deaths per 100,000 population per year, respectively; Vardoulakis et al., 2014). Consequently, in 2014 there were over 16,000 hospital admissions in the UK whereby hypothermia was the primary or secondary cause (HSCIC, 2015). Exposure to environments beneath thermoneutrality clearly present a major health risk, particularly in thermoregulatory vulnerable populations such as the very young and the elderly, who account for more than 85% of these admissions. The primary deleterious effects of cold on the human body arise when T_C_ falls below 35°C, although the autonomic physiological responses required to maintain T_C_ (tachycardia and shivering) can increase cardiovascular strain and can lead to secondary events [particularly in elderly individuals (Parsons, 2014)]. The data obtained in this experiment demonstrates that T_C_ is not as efficiently defended when participants ingest APAP in a subneutral environment. This is particularly concerning as the average thermal sensation in both groups (APAP and placebo) was 3.4 ± 2 (3.5 = “comfortable”), and no participants reported feeling “cold” on the thermal sensation scale. If APAP inhibited normal thermogenic mechanisms in these conditions, it is likely that these T_C_ reductions will be exacerbated in colder environments. More work is needed in this area to confirm when the peak reduction in T_C_ arises, and the variability in this response. Moreover, it is unclear if APAP induced hypothermia is exacerbated in cold conditions (where there is a greater reliance on thermogenesis), which may be inhibited in the presence of a COX inhibitor (such as APAP). This specific hypothesis has recently been proposed by our group (Foster et al., 2015), and should be investigated in future work. These findings could have implications for public health recommendations.

The molecular target for APAP induced hypothermia in humans is not well established, but is likely due to inhibition of the COX enzyme. When administered orally (1000 mg), APAP is a potent inhibitor of COX-2 in intact cells, but may also inhibit COX-1 (Hinz et al., 2008). Given that participants in this study were exposed to sub-neutral environmental temperatures, it is possible that COX may have been prevented from activating thermogenic responses to this environment (Foster et al., 2015). Although the role of COX in non-febrile thermogenesis remains a topic of debate (Aronoff and Romanovsky, 2007; Foster et al., 2015), recent evidence supports a role for COX in this capacity. For example, it has been demonstrated that COX-2 is essential for UCP-1 induction in beige/brite adipocytes during cold exposure; T_C_ was not efficiently defended during acute cold exposure in COX-2 gene deficient mice compared with their wild-type counterparts (Madsen et al., 2010). More recently, intravenous parecoxib (COX-2 selective inhibitor) administration significantly reduced post-operative shivering in non-febrile patients (Li et al., 2014; Shen et al., 2015). Thus, it is possible that during sub neutral conditions, a reduction in autonomic shivering responses (mediated by APAP induced COX-2 inhibition) could contribute to the decline in T_C_ witnessed in the present work (**Figure 3**).

## Conclusion

It has been demonstrated that acute APAP ingestion at a dose of 20 mg⋅kg lean body mass reduces non-febrile T_C_ during a 120 min passive exposure to 20°C, 40% r.h (**Figure 3**). Future research should seek to determine if APAP reduces the capacity of the thermoregulatory system to maintain T_C_ during cold exposure. Moreover, it should be determined if the peak reductions in T_C_ are in line with peak CSF concentrations. Such findings would determine (i) if APAP induced hypothermia is mediated through a brain derived mechanism and (ii) if these T_C_ reductions have implications for the pathology of accidental hypothermia.

## Author Contributions

JF, LT, and AM contributed to the study design, data interpretation and manuscript revision. JF, KT, and SW contributed to data collection and also contributed to manuscript revision. All aspects of the project were supervised by LT, and AM. All authors approved the final version of the manuscript and all authors qualifying for authorship are listed.

## Conflict of Interest Statement

The authors declare that the research was conducted in the absence of any commercial or financial relationships that could be construed as a potential conflict of interest.
