# Corrigendum: Effect of Acetaminophen Ingestion on Thermoregulation of Normothermic, Non-febrile Humans

**DOI:** 10.3389/fphar.2016.00093

**Published:** 2016-04-05

**Authors:** Josh Foster, Alexis Mauger, Katie Thomasson, Stephanie White, Lee Taylor

**Affiliations:** ^1^Applied Sport and Exercise Physiology Research Group, Department of Sport Science and Physical Activity, Institute of Sport and Physical Activity Research, University of BedfordshireBedford, UK; ^2^Endurance Research Group, School of Sport and Exercise Sciences, University of Kent, Chatham MaritimeKent, UK; ^3^ASPETAR, Qatar Orthopaedic and Sports Medicine Hospital, Athlete Health and Performance Research CentreDoha, Qatar

**Keywords:** acetaminophen, paracetamol, tylenol, thermoregulation, temperature, therapeutichypothermia

Due to a misunderstanding, the requirements of the funding institution, Aspetar, regarding the affiliation order were not followed during production. In the original article, Lee Taylor's affiliation was affiliated with institutions “1,3” in that order.

According to the requirements of the funding institution, Dr. Taylor should be affiliated with Aspetar first and so it should be “3,1.”

The authors apologize for this. This error does not change the scientific conclusions of the article in any way.

The original article has been updated.

## Author contributions

JF, LT, and AM contributed to the study design, data interpretation and manuscript revision. JF, KT, and SW contributed to data collection and also contributed to manuscript revision. All aspects of the project were supervised by LT, and AM. All authors approved the final version of the manuscript and all authors qualifying for authorship are listed.

### Conflict of interest statement

The authors declare that the research was conducted in the absence of any commercial or financial relationships that could be construed as a potential conflict of interest.

